# Active Gaze Guidance and Pupil Dilation Effects Through Subject Engagement in Ophthalmic Imaging

**DOI:** 10.3390/jemr18050045

**Published:** 2025-09-19

**Authors:** David Harings, Niklas Bauer, Damian Mendroch, Uwe Oberheide, Holger Lubatschowski

**Affiliations:** 1 Institute of Applied Optics and Electronics, TH-Köln—University of Applied Sciences Research, 50679 Cologne, Germany; 2 OCUMAX Healthcare GmbH, 30419 Hannover, Germany

**Keywords:** gaze guidance, pupil dilation, eye tracking, fundus imaging, optical coherence tomography, gamification

## Abstract

Modern ophthalmic imaging methods such as optical coherence tomography (OCT) typically require expensive scanner components to direct the light beam across the retina while the patient’s gaze remains fixed. This proof-of-concept experiment investigates whether the patient’s natural eye movements can replace mechanical scanning by guiding the gaze along predefined patterns. An infrared fundus camera setup was used with nine healthy adults (aged 20–57) who completed tasks comparing passive viewing of moving patterns to actively tracing them by drawing using a touchpad interface. The active task involved participant-controlled target movement with real-time color feedback for accurate pattern tracing. Results showed that active tracing significantly increased pupil diameter by an average of 17.8% (range 8.9–43.6%; *p* < 0.001) and reduced blink frequency compared to passive viewing. More complex patterns led to greater pupil dilation, confirming the link between cognitive load and physiological response. These findings demonstrate that patient driven gaze guidance can stabilize gaze, reduce blinking, and naturally dilate the pupil. These conditions might enhance the quality of scannerless OCT or other imaging techniques benefiting from guided gaze and larger pupils. There could be benefits for children and people with compliance issues, although further research is needed to consider cognitive load.

## 1. Introduction

Modern ophthalmic imaging techniques such as funduscopy and optical coherence tomography (OCT) are vital tools in diagnosing and monitoring eye diseases like glaucoma and macular degeneration. However, examinations often rely on passive patient behavior, which can lead to issues such as eye fatigue, frequent blinking, and difficulties maintaining a stable gaze. In addition, patient discomfort and lack of engagement can have a negative impact on the quality of images, leading to longer examination times.

Traditional OCT devices typically require patients to maintain a passive, fixed gaze while a scanning mirror moves the OCT beam across the retina to capture detailed images. Our approach in the FALCO (Fourier Algorithm-Based Low-Cost OCT) project aims to develop a cost-effective glaucoma screening device that eliminates the need for built-in scanner hardware by replacing it with patient-driven gaze guidance. In this concept, the patient’s eye follows predefined scan paths on a display, effectively performing the scanning motion while the OCT beam remains fixed. A fundus-based eye tracker was implemented, which determines gaze position by continuously registering retinal markers in real time and can therefore assign the OCT measurement points. Unlike conventional OCT or existing eye tracking systems, which are primarily used for motion correction, this method actively uses natural eye movements as a scanning mechanism. This setup has already been described in detail in previous publications [1,2,3].

This paper presents a gamification approach for ophthalmic imaging in which patients actively follow and trace visual patterns during the examination. Here, gamification means integrating structured, goal-oriented tasks that keep the patient’s attention focused and improve cooperation throughout the procedure. During the exam, the patient’s gaze is guided by visual patterns displayed on a screen, and the patient uses a touchpad to trace these patterns manually. This engagement technique not only promotes active involvement but has also been observed to increase pupil dilation, minimize blinking, and reduce involuntary eye movements, improving image quality.

Hess and Polt [4] conducted a study in the early 1960s in which they found that changes in pupil size during the solution of simple multiplication tasks can serve as a direct indicator of mental activity. The more difficult the task, the greater the pupil dilation. These findings were supported by subsequent studies. Kahneman and Beatty [5] discovered that there is a clear correlation with memory load when memorizing numbers. They showed that this cognitive load influences pupil size. The more complex the cognitive task, the greater the pupil dilation. This was confirmed by further research by Ahern and Beatty [6] and Wagoner [7].

Richer and Beatty [8] extended this knowledge by showing that motor processing demands both before and after a movement have a significant influence on pupil response. Their study showed that pupils dilate during finger movements, illustrating the relationship between motor activity and pupil size. These and many other experiments have been summarized in 2000 in a comprehensive literature review and confirm the general finding that memory load and motor processing have a significant enlarging effect on pupil size [9].

Recent comprehensive reviews by van der Wel and van Steenbergen [10] have summarized extensive evidence that task-evoked pupil dilation is a robust psychophysiological marker of cognitive effort and task engagement. These findings support the theoretical basis for our approach, which builds on the established link between increased cognitive load and pupil dilation. In addition, it is documented that the pupil responds not only to cognitive effort but also to emotional arousal, stress, and sympathetic nervous system activation, as well as motivational factors, all of which can play a role in gamified tasks [11,12,13,14,15].

Taken together, this research indicates that pupil dilation is a robust and sensitive marker of both cognitive effort and emotional arousal. These mechanisms can be used in ophthalmic examinations to promote stable following of the fixation target, reduce blinking, and naturally enlarge the pupil through structured patient engagement.

## 2. Method

The original setup for OCT-imaging consisted of an infrared fundus camera and a fixation target presented on a display. For this experiment, the fundus camera system was modified to image the pupil instead of the retina to analyze changes in pupil size during different tasks. The subject viewed this target with the tested eye while the pupil was recorded. To minimize accommodation effects, the display image was inserted into the optical path via a beam splitter and optically projected to appear at a far distance to the viewer. Targets were presented on a 2.1-inch TFT-LCD (1600 × 1600 px, 90 Hz). Brightness and contrast of the display were constant across all conditions. Nine test subjects (aged 20–57) were examined through the system.

All measurements were performed in a darkened room. Before the experiment started, participants received detailed instructions while their eyes adapted to the low-light conditions. Participants were given one to two minutes to adapt to the darkened environment before the examinations began, ensuring that the pupil diameter reached a stable maximum baseline.

Participants controlled a target on the display using a touchpad. In pilot testing, various input devices (joystick, trackball, touchpad) were evaluated for usability and motion artifacts. The touchpad was selected because it caused the least arm movement and was easiest for participants to use.

**Figure 1:**
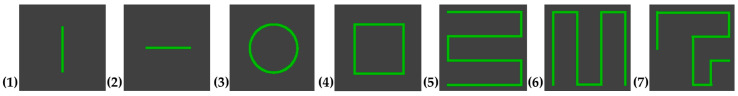
Various pattern options: (1) vertical line, (2) horizontal line, (3) circle, (4) square, (5) horizontal grid, (6) vertical grid, and (7) complex zigzag pattern.

A custom program displayed the seven predefined visual patterns shown in Figure 1. Each pattern was drawn within a defined area on the screen. A small green square (5 × 5 pixels) served as the controllable target. The patterns were shown and then had to be traced by drawing on the blank screen. The target remained green as long as the subjects were on the correct path. If they deviated, the color of the target changed to blue to indicate that they were outside the intended path. The path itself had a certain tolerance. It was sufficient for the target to touch the intended path with just one pixel for it to remain green. This immediate visual feedback helped participants adjust their tracing accuracy in real time. As soon as the test subjects traced the path correctly, meaning that the majority of the path coordinates were hit, the program confirmed the successful completion at the target position by flashing visually.

**Figure 2:**
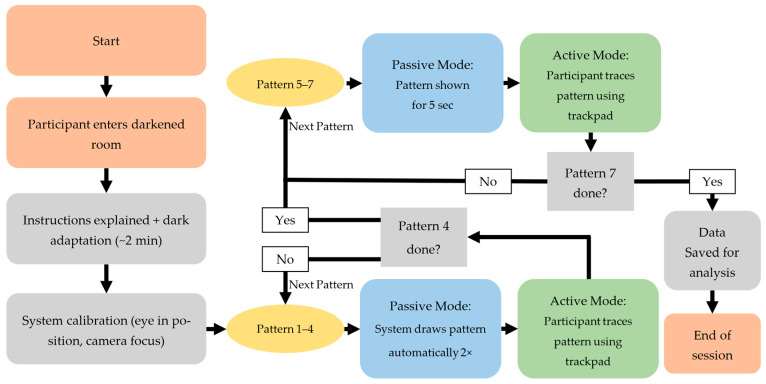
Schematic flowchart of the experimental procedure.

The experimental procedure is illustrated in Figure 2.

**Figure 3:**
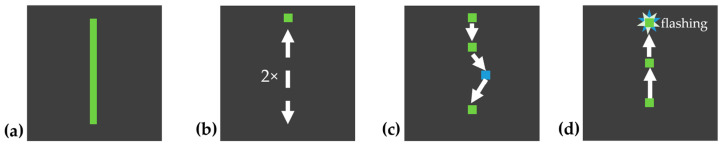
Example pattern 1: (**a**) pattern path; (**b**) automatically moving target shown to participants (2×); (**c**) participant drawing the path with color feedback (blue if off-path); and (**d**) correct tracing confirmed by flashing. The arrows indicate the direction of target movement, the squares mark key positions along the path, and the stars represent the flashing confirmation signal.

The experiment was divided into two modes, as shown in Figure 2. Patterns 1–4 were demonstrated by the system, with an automatically moving target tracing the pattern path. The moving target traced the pattern from the start point to the end point and back to the start point twice at a steady speed, to allow the participant to observe the full path as shown in Figure 3b. After the demonstration, the target returned to the start position and the participant was instructed to draw the same pattern themselves, point by point, using the touchpad, as shown in Figure 3c. Patterns 1 and 2 were simple and served as training to familiarize the participant with the system. Patterns 3 and 4 were slightly more complex to increase cognitive load. The complexity of each pattern was determined based on the number of directional changes, the geometric shape, whether the entire pattern was visible, and the cognitive load required (simple shapes vs. complex, memory-dependent structures). Based on these criteria, patterns 1 and 2 were classified as simple, patterns 3 and 4 as moderately complex, and patterns 5 and 7 as complex.

**Figure 4:**
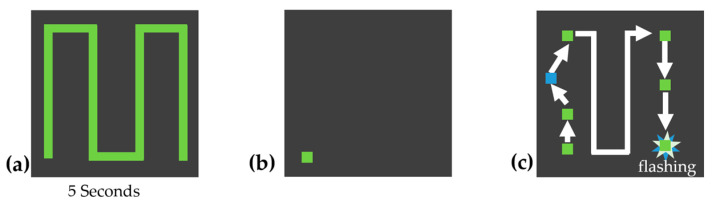
Example pattern 5: (**a**) pattern shown to participants for 5 s; (**b**) target jumps to start position; and (**c**) participant traces the pattern from memory. The arrows indicate the direction of target movement, the squares mark key positions along the path, and the stars represent the flashing confirmation signal.

Patterns 5–7 were shown as static images for 5 s. Participants could view the full pattern at once, as shown in Figure 4. Afterwards, the controllable target automatically reset to the start position, and participants were asked to reproduce the pattern from memory. Patterns 5 and 6 resembled typical line-scan structures in OCT imaging, while pattern 7 was designed to be more complex, aiming to maximize cognitive load and pupil response. All subjects performed the same sequence of patterns.

Before starting each measurement, the center of the pupil was manually centered in the camera image and the focus was checked. The position was regularly checked during recording to ensure consistent accuracy.

During each trial, the participant’s pupil size and blink frequency were recorded using a monochrome CMOS camera (model U3-3180CP-M-GL, IDS Imaging Development Systems GmbH, Obersulm, Germany). The camera operated at its maximum frame rate of 73 fps under constant infrared illumination with 2592 × 2048 pixels.

The pupil diameters were extracted frame by frame using a custom Python script (Python 3.11.9, OpenCV-based segmentation, Hough circle detection, smoothing across multiple frames). All automatically determined values were manually verified, and incorrectly detected measurements, mainly due to jerky head movements or blinks, were discarded (14.48% of data points). Blink detection relied on brightness changes in the pupil area and was manually validated, showing high agreement with the visual check. For calibration, a reference object of 5 mm diameter was recorded, corresponding to 280 pixels in the video (conversion factor: 5 mm/280 px ≈ 0.0179 mm/px). The camera image of the eye was recorded together with the displayed stimulus in a combined 1920 × 1080 video with OBS Studio. Since the high-resolution camera images (2592 × 2048 px) were downscaled and embedded into the combined 1920 × 1080 video stream, the calibration factor was derived from this compressed recording. For the pupil size analysis, only the region of the video containing the eye was considered.

For each pattern, the two conditions were compared in terms of pupil size and blink frequency to assess differences between passive viewing and active engagement.

All subjects participated with their best optical correction. Two subjects wore spectacles and one subject wore contact lenses during the experiment, while the remaining participants had sufficient uncorrected visual acuity.

The proof-of-concept comprises a total of 9 subjects, 8 of whom are male and 1 female, aged 20 to 57 years shown in Table 1.

## 3. Results

### 3.1. Pupil Dilation

The direct comparison of pupil sizes during passive viewing and active tracing by drawing reveals a consistent increase in pupil diameter when subjects actively trace the pattern. In nearly all cases, the pupil dilated noticeably at the start of the interactive task and remained larger throughout the active condition compared to passive observation of the same pattern. This supports the hypothesis that active engagement and motor-cognitive interaction lead to measurable physiological effects on the pupil.

**Figure 5:**
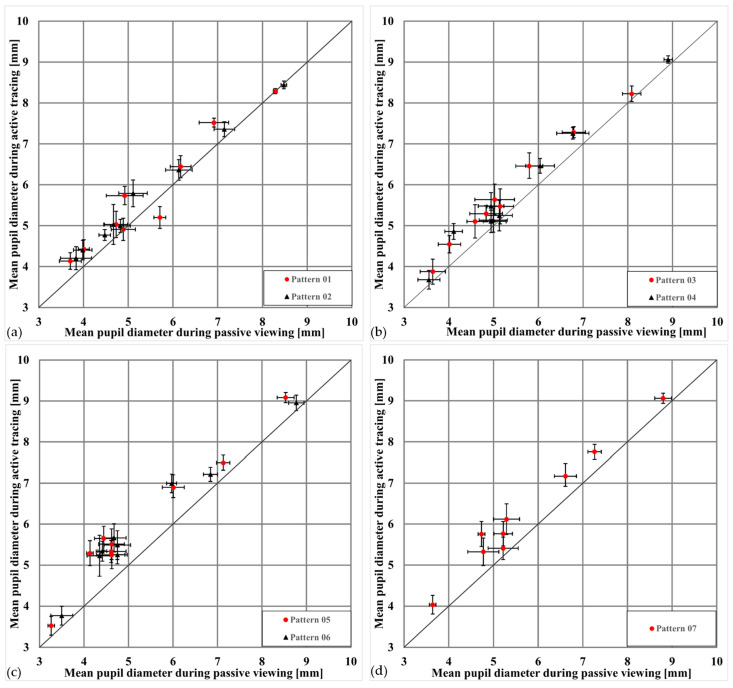
Comparison of mean pupil diameters during viewing and drawing of different patterns. (**a**) The mean pupil diameter distribution when viewing (x-axis) and drawing (y-axis) patterns 01 (vertical line) and 02 (horizontal line). The points mostly lie just above the equality line [(3, 3), (10, 10)], indicating a small effect of these patterns on pupil size. (**b**) The pupil diameters for patterns 03 (circle) and 04 (square). Here, the effect is more pronounced, with more points lying significantly above the equality line, indicating a greater pupil dilation while drawing these patterns. (**c**) Presents data for patterns 05 (horizontal grid) and 06 (vertical grid). Subjects viewed the patterns briefly for 5 s and then drew them. Results show an even larger effect, with increased pupil dilation, suggesting that higher cognitive demands cause greater pupil enlargement. (**d**) Pattern 07 (complex zigzag), intentionally difficult to remember. The pupils show the greatest effect, as the pupil is significantly larger during drawing compared to viewing, reflecting the high cognitive demands.

**Figure 6:**
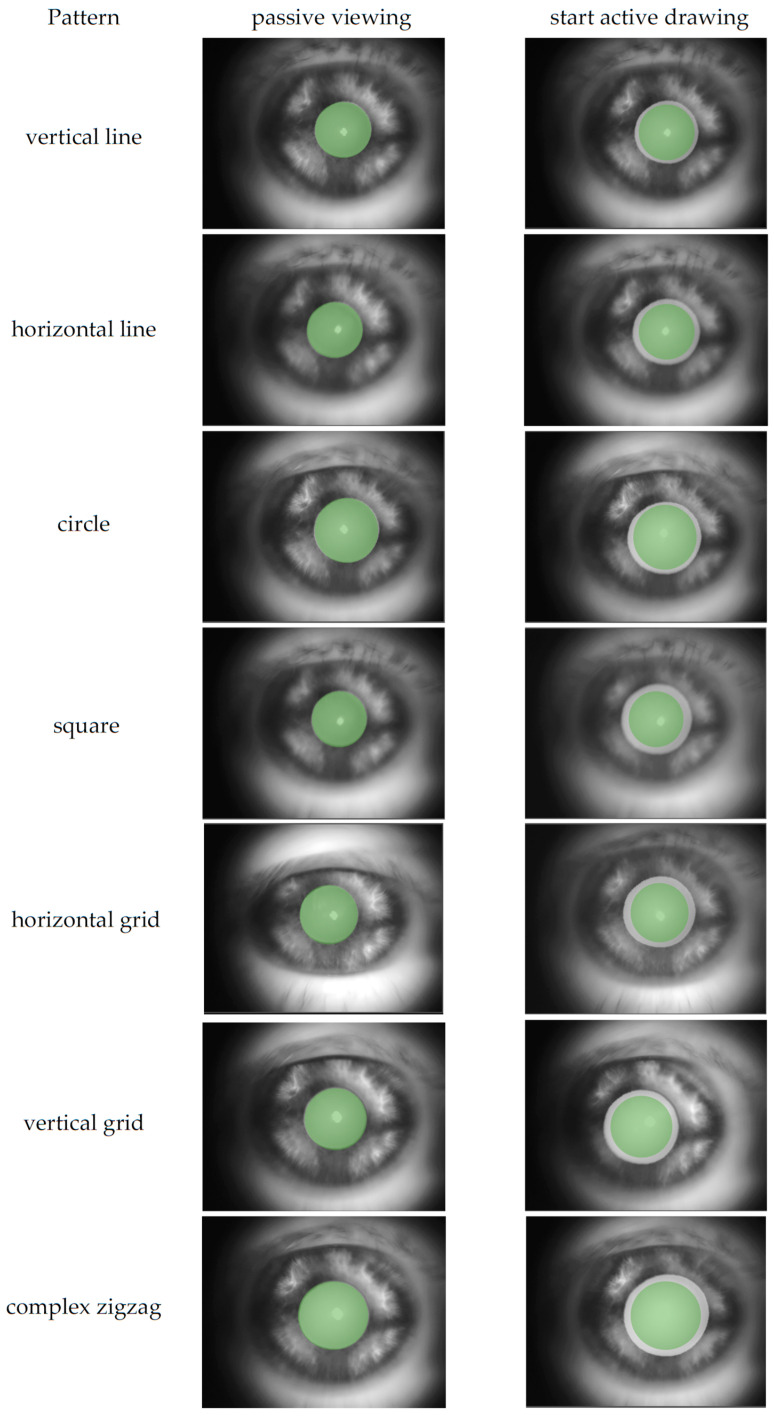
Example pupil diameter of one subject for all patterns. The left column shows the pupil size during passive viewing; the right column shows the pupil size at the start of active tracing by drawing. The green overlay represents the passive viewing pupil diameter and is overlaid onto the active tracing image for direct comparison. This highlights the typical immediate pupil dilation when the participant takes control of the tracing task.

Figure 5 summarizes this effect across all patterns and participants, showing that more complex patterns and memory-based tasks amplify the dilation effect. Pupil size values represent mean pupil diameters (in mm) calculated across all valid frames within each condition and pattern for each subject. To illustrate this visually, Figure 6 presents an example from one subject highlighting the typical change in pupil size between passive and active conditions for the same pattern.

In all diagrams shown in Figure 5, most data points lie above the equality line, consistently showing that pupil diameters are larger when drawing the patterns than when merely viewing them. This supports the hypothesis that active engagement and associated cognitive activity lead to greater pupil dilation.

To validate these observations statistically, a linear mixed model (LMM) with Subject as a random effect revealed a significant main effect of condition (viewing vs. drawing) on mean pupil diameter, F(1,110) = 102.42, *p* < 0.001, indicating bigger pupil sizes during active drawing compared to passive viewing. In addition, there was a significant main effect of pattern complexity, F(6,110) = 6.31, *p* < 0.001, suggesting that more complex or memory-based patterns elicited dilation responses. These findings confirm that both task engagement and stimulus complexity reliably modulated pupil size across subjects. Table 2 shows the descriptive statistics of pupil diameter by condition.

**Figure 7:**
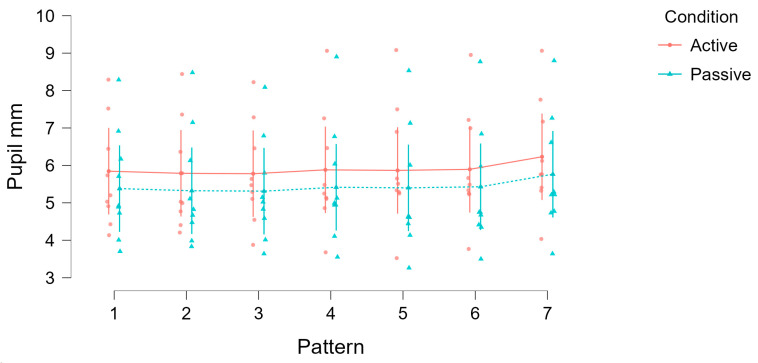
Pupil diameters across conditions (active vs. passive) separated by pattern complexity. Lines represent mean values for each pattern and error bars indicate standard errors of the mean.

Figure 7 visualizes these results across subjects and patterns. In all cases, mean pupil diameters were greater in the active condition than in the passive condition. Error bars reflect standard errors of the mean, which appear relatively large due to substantial inter-individual differences in baseline pupil size. Nevertheless, the consistent shift between conditions demonstrates that both task engagement and stimulus complexity reliably modulated pupil responses.

To further illustrate this effect, the mean pupil diameter for each subject when passively viewing the simplest pattern (pattern 1) and actively drawing the most complex pattern (pattern 7) was compared in Table 3. The results show that pupil diameter increased by an average of ~18% (range 9–44%), supporting the finding that higher cognitive demands and motor engagement lead to consistent pupil enlargement.

An average increase in pupil diameter of ~18% translates into a substantially larger pupil area, as the area is proportional to the square of the diameter. This enlargement allows significantly more light to reach the retina, which is highly relevant for optimizing image quality in scannerless OCT and similar applications.

Further details and additional information, including a sample video showing the pupil and the pattern during passive viewing and active tracing, can be found in the Supplementary Materials.

### 3.2. Blink Rate

In addition to measuring pupil diameter, the blink behavior of the subjects was also examined. Blinking is a natural and involuntary action that can influence the quality of ophthalmic imaging by temporarily obscuring the pupil. During the experiment, the number of blinks was recorded while subjects viewed and drew the various patterns. A blink was defined as any interruption in the visibility of the pupil, including rapid eyelid movements. Table 4 presents the summary of blink counts for each subject during the viewing and drawing phases of the experiment, along with the total time spent in each condition.

The frequency of blinks varied greatly between subjects. For example, subject 1 had no blinks in either condition, while subject 9 had a total of 35 blinks when viewing and 14 when drawing. The difference in blink frequency (Table 4 blink difference) indicates that drawing the patterns tends to lead to a reduction in blink frequency compared to viewing, as was observed in most subjects.

Despite the longer time needed to draw the patterns compared to viewing them, the frequency of blinking was lower in most cases. This is clearly indicated by the “Blink difference” column in the table. For example, subject 4 showed 29 blinks during viewing, but only 2 blinks during drawing, even though the drawing duration was more than twice as long. The reduced blink rate during the longer drawing phases could indicate that the active participation through drawing increases the visual and cognitive attention of the test subjects, which leads to fewer blinks.

Blink counts were analyzed using a generalized linear mixed model (GLMM) with condition (viewing vs. drawing) and Pattern complexity (Patterns 1–7) as fixed effects, and Subject as a random intercept (Table 5). The analysis revealed a significant main effect of condition on blink counts, χ^2^(1) = 39.24, *p* < 0.001, indicating that subjects blinked significantly less during active drawing compared to passive viewing. There was also a significant main effect of pattern, χ^2^(6) = 101.61, *p* < 0.001, showing that blink rates varied systematically with stimulus complexity.

**Figure 8:**
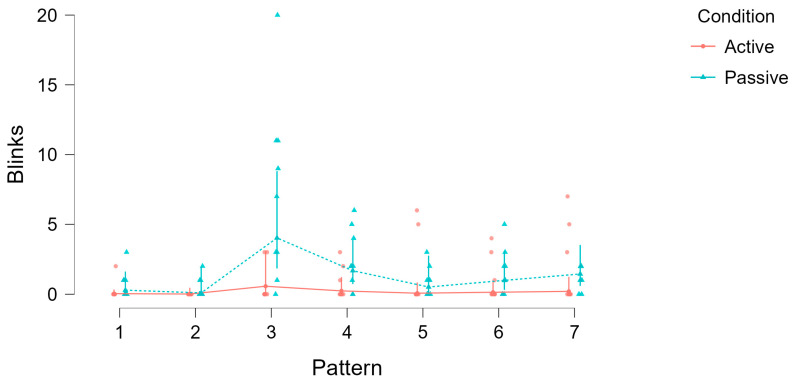
Blink counts across patterns (1–7) for active (drawing) and passive (viewing) conditions. Dots represent individual data points, lines show condition means, and error bars indicate standard errors. Despite longer durations during drawing, blink frequency was consistently lower in the active condition compared to passive viewing, and blink rates varied with pattern complexity.

To illustrate these effects, Figure 8 visualizes blink counts across all patterns for both conditions. The plot shows that, despite the longer duration of the drawing phases, blink frequency was consistently lower in the active condition compared to passive viewing.

### 3.3. Eye Movement

In addition to pupil diameter and blinking behavior, eye movements were qualitatively assessed based on the recorded video data. Visual inspection indicated that gaze trajectories appeared noticeably smoother and more stable during active pattern tracing compared to passive viewing. However, since the positional tracking data require further quantitative analysis, these observations should be considered preliminary.

### 3.4. Subjective Feedback

Many of the subjects reported that the gamified approach to ophthalmic imaging was not only engaging but also enjoyable. A common theme in their feedback was the sense of challenge and achievement they experienced while attempting to accurately trace the patterns. This gamified interaction fostered a degree of personal investment and competitiveness, motivating participants to improve their performance with each attempt.

Additionally, the participants expressed that the active gaming aspect of the examination was significantly more pleasant and less tedious than the traditional method of passively staring at the system. The interactive nature of the tasks kept them more focused and alert, reducing the discomfort and fatigue often associated with prolonged passive fixation. Overall, this suggests that such an approach could improve collaboration and patient experience in the clinical setting.

No subject reported fatigue or difficulties; however, it is possible that more complex patterns in larger studies could lead to increased fatigue, which should be systematically investigated. The subjective feedback was collected informally and not systematically, which is a limitation. Structured collection using standardized questionnaires is planned for future work.

## 4. Discussion

This proof-of-concept study demonstrates that gamified patient engagement during ophthalmic imaging can measurably affect physiological parameters such as pupil diameter and blink frequency. Compared to passive viewing, actively tracing by drawing visual patterns led to a consistent increase in pupil diameter (average ~18% increase) and a reduction in blink frequency across subjects. This aligns with previous findings that link cognitive and motor engagement to increased pupil dilation.

The present study is exploratory and due to the small number of participants (n = 9) and the unbalanced gender distribution (8 male, 1 female), the results are only transferable to the general population to a limited extent. This represents a significant limitation that should be considered when interpreting the results. Future studies should include larger and more diverse samples with balanced representation across gender and age groups. Such follow-up work will be essential to determine whether the observed effects can be generalized beyond this proof-of-concept study.

By encouraging natural pupil dilation through interactive tasks, this approach might have the potential to reduce pharmacological dilation with mydriatic eye drops, which often causes side effects like light sensitivity and blurred vision and requires significant waiting time. The approach is intended for routine use and simple examinations and might facilitate rapid screening, including in mobile or resource-limited settings. While pharmacological dilation can last 10 to 40 min or for several hours (or even days for certain substances) [16,17,18,19], cognitive-driven dilation could allow imaging to begin immediately, improving patient comfort and examination efficiency. However, the effect is likely to vary from person to person and may be insufficient in some population groups, meaning that cognitively induced pupil dilation should not yet be considered a complete replacement for pharmacological mydriasis.

In our scannerless OCT concept, the patient’s natural eye movements are used to replace expensive scanning hardware, and the observed pupil dilation emerges as an additional beneficial side effect of this interactive guidance. Importantly, the principle of gamified patient engagement could also be applied to other ophthalmic procedures beyond OCT, such as fundus imaging, visual field testing, or even pupillometry itself, where improved cooperation and natural dilation may enhance diagnostic quality.

A limiting factor of the developed gamification approach is that the pupil still reacts to visible light stimuli, which is why an environment with reduced lighting is required to maximize pupil dilation through cognitive stimulation. Therefore, the fundus examination is performed using infrared illumination. This allows the fundus to be imaged in our setup without the pupil reacting to the light.

The use of gamification seems particularly useful for special patient groups, such as children or patients with limited compliance, for example, in the case of neurological diseases. In such cases, the playful component could significantly increase cooperation and patient engagement.

Although gamification offers promising advantages in the context of this study, potential disadvantages should not be ignored. For example, some patients may experience cognitive overload, particularly with complex tasks, which in some cases may not lead to sufficient pupil dilation. This was not considered further and can be analyzed in subsequent studies, along with the open question of whether cognitive or motor engagement has the predominant influence on the effect.

The integration of gamification techniques in ophthalmic imaging represents a promising innovation in enhancing patient engagement and diagnostic efficacy. The findings from our proof-of-concept study demonstrate the physiological and practical benefits of actively involving patients in their eye examinations. The significant pupil dilation observed during the active tasks aligns with the literature, supporting the idea that increased cognitive engagement leads to enhanced pupillary response. Moreover, the reduction in blink rate and potential stabilization of eye movements further contribute to the quality of the diagnostic images obtained. The tear film becomes thinner with longer measuring times and little to no blinking, which could lead to a different type of artifact—a disadvantage that was not further investigated in this study and has to be addressed in further studies. In particular, tear film properties are known to vary with gender and age [20,21,22], which makes it important to examine resulting artifacts, their impact on image quality, and the associated deterioration of corneal optical quality in subsequent studies.

These improvements address several limitations inherent in traditional passive imaging methods, such as eye fatigue, frequent blinking, and unstable gaze, which often compromise image quality and prolong examination times. By transforming the examination into an interactive experience, patients not only become more invested in the process but also exhibit physiological responses that are conducive to better imaging outcomes. At least in the developed OCT system, a larger pupil further enlarges the effective aperture, thereby increasing the retinal area accessible through gaze-guided scanning and improving overall fundus image quality.

While the initial results are promising, further research is required to fully understand the benefits and limitations of gamified ophthalmic imaging. Specifically, the preliminary analysis of eye movement stability suggests smoother movements during active engagement, but a more detailed analysis is necessary to substantiate this finding. Future work will include a quantitative assessment of gaze stability using objective measures such as position variance or fixation stability metrics. This will allow us to verify whether the qualitative changes observed during active tracking lead to measurable improvements. For further tests, the system will be reverted to its original state of looking at the retina rather than the pupil to allow more precise tracking of eye movements during various tasks.

One other limitation of the present proof-of-concept study is that the active condition introduced additional factors besides the intended cognitive involvement, including motor actions (touchpad input) and visual feedback through color changes. These aspects can independently influence pupil size and blink rate and were not systematically controlled in the current design. Future studies should therefore consider specific control conditions, such as motor tasks without memory requirements or passive viewing with visual feedback, in order to distinguish these influencing factors from one another.

While our findings are consistent with a cognitive-effort interpretation, pupil dilation is not a unique marker of mental load. Emotional arousal, stress, and sympathetic nervous system activation can also affect pupil size [13,15]. Since no auxiliary physiological measures (e.g., heart rate, skin conductance) or subjective workload ratings were collected in this study, these alternative drivers cannot be disentangled from the observed effects. This limitation should be considered when interpreting the present results, and future studies should therefore combine pupillometry with multimodal physiological and behavioral measures to more precisely attribute pupil changes to cognitive engagement.

Additionally, expanding the participant pool to include a broader demographic range will help generalize the findings and determine the approach’s efficacy across different age groups. Another area of future research could involve exploring the integration of gamified tasks with other imaging modalities, broadening the scope and applicability of this approach.

## Figures and Tables

**Table 1:** Demographic distribution of subjects, including gender and age range.

Parameter	Subjects
*n*	9
Male	8
Female	1
Age range (y)	20–57

**Table 2:** Summary from the linear mixed model (Type III ANOVA, Satterthwaite’s method) showing the effects of condition and pattern on pupil diameter.

Effect	df	F	*p*
Condition	1110.00	102.419	<0.001
Pattern	6110.00	6.310	<0.001

**Table 3:** Comparison of mean pupil diameters (in mm) for all nine subjects during passive viewing (pattern 1) and active tracing (pattern 7). Shown are the mean values for each condition and the absolute difference, with the percentage change relative to passive viewing.

Subject	Mean Pupil Diameter Viewing Pattern 1 [mm]	Mean Pupil Diameter Drawing Pattern 7 [mm]	Absolute Difference [mm]	Percentage Increase [%]
1	8.287	9.064	0.777	9.37
2	4.888	5.324	0.435	8.91
3	6.171	7.170	0.999	16.19
4	6.916	7.757	0.841	12.16
5	4.921	5.764	0.843	17.134
6	4.728	5.764	1.037	21.93
7	5.087	6.120	1.033	20.31
8	4.007	5.756	1.749	43.639
9	3.704	4.034	0.330	8.921

**Table 4:** Summary of blink counts for each subject during viewing and drawing of patterns, the difference between these conditions and the blinks per second.

Subject	Total Blinks Viewing	Total Blinks Drawing	Difference	Total Time Viewing [s]	Total Time Drawing [s]	Blinks per Second Viewing	Blinks per Second Drawing
1	0	0	0	101	284	0	0
2	23	22	1	101	178	0.2277	0.1236
3	4	0	4	101	148	0.0396	0
4	29	2	27	101	217	0.2871	0.0092
5	16	6	10	101	198	0.1584	0.0303
6	12	3	9	101	219	0.1188	0.0137
7	4	0	4	101	177	0.0396	0
8	11	1	10	101	184	0.1089	0.0054
9	35	14	21	101	214	0.3465	0.0654

**Table 5:** Summary of the statistical parameters for the generalized linear mixed model (GLMM) with Poisson distribution and log link function. Reported are the effects of condition (viewing vs. drawing) and pattern complexity on blink counts, with degrees of freedom (df), Chi-square values (χ^2^), and significance levels (*p*).

Effect	df	χ^2^	*p*
Condition	1	39.24	<0.001
Pattern	6	101.61	<0.001

## Data Availability

The data presented in this study are available on request from the corresponding author due to confidentiality and privacy disclosure of the participants’ identities and authorized sharing of the data.

## Supplementary Materials

The following supporting information can be downloaded at: https://www.mdpi.com/article/10.3390/jemr18050045/s1, Video S1: example_video.mp4, LMM Data Pupil Dilation: Pupil_Dilation.csv, GLMM Data Blinks: Blinks.csv.
