# Supplementary material for: Active Gaze Guidance and Pupil Dilation Effects Through Subject Engagement in Ophthalmic Imaging
Source: J Eye Mov Res. 2025 Sep 19;18(5):45. doi: 10.3390/jemr18050045 (PMC12452769; doi:10.3390/jemr18050045)
Supplement: Supplementary file 1 [file jemr-18-00045-s001.zip › Blinks.html]

JASP 


# Results

## Generalized Linear Mixed Models

| ANOVA Summary | | | | | | | |
| --- | --- | --- | --- | --- | --- | --- | --- |
| Effect | | df | | ChiSq | | p | |
| Condition |  | 1 |  | 39.24 |  | < .001 |  |
| Pattern |  | 6 |  | 101.61 |  | < .001 |  |
|  | | | | | | | |
|  |  |  |  |  |  |  |  |
| --- | --- | --- | --- | --- | --- | --- | --- |
| *Note.*  Generalized linear mixed model with poisson family and log link function. | | | | | | | |
| *Note.*  Model terms tested with likelihood ratio tests testMethod. | | | | | | | |
| *Note.*  The following variable is used as a random effects grouping factor: 'Subject'. | | | | | | | |
| *Note.*  Type III Sum of Squares | | | | | | | |

### Plot

| Estimated Marginal Means | | | | | | | | | |
| --- | --- | --- | --- | --- | --- | --- | --- | --- | --- |
|  | | | | | | 95% CI | | | |
| Condition | | Estimate | | SE | | Lower | | Upper | |
| Active |  | 0.327 |  | 0.145 |  | 0.137 |  | 0.780 |  |
| Passive |  | 0.884 |  | 0.380 |  | 0.381 |  | 2.053 |  |
|  | | | | | | | | | |
|  |  |  |  |  |  |  |  |  |  |
| --- | --- | --- | --- | --- | --- | --- | --- | --- | --- |
| *Note.*  Results are averaged over the levels of: Pattern. | | | | | | | | | |
| *Note.*  Results are on the response scale. | | | | | | | | | |
