# Supplementary material for: Active Gaze Guidance and Pupil Dilation Effects Through Subject Engagement in Ophthalmic Imaging
Source: J Eye Mov Res. 2025 Sep 19;18(5):45. doi: 10.3390/jemr18050045 (PMC12452769; doi:10.3390/jemr18050045)
Supplement: Supplementary file 1 [file jemr-18-00045-s001.zip › Blinks.pdf]

Results

Generalized Linear Mixed Models

ANOVA Summary

Effect	df	ChiSq	p
Condition	1	39.24	< .001
Pattern	6	101.61	< .001

Note. Generalized linear mixed model with poisson family and log link function.
Note. Model terms tested with likelihood ratio tests testMethod.
Note. The following variable is used as a random effects grouping factor: 'Subject'.
Note. Type III Sum of Squares

Plot

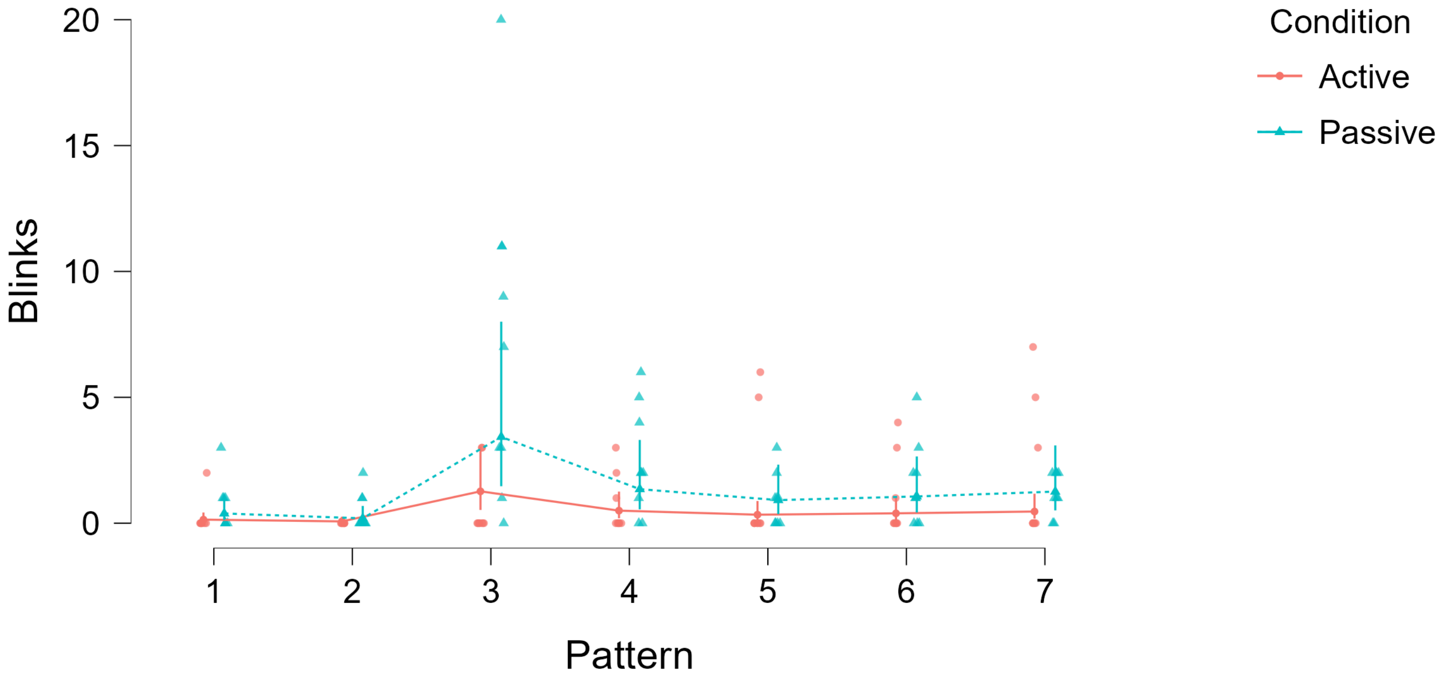

Estimated Marginal Means

			95% CI	
Condition	Estimate	SE	Lower	Upper
Active	0.327	0.145	0.137	0.780
Passive	0.884	0.380	0.381	2.053

Note. Results are averaged over the levels of: Pattern.
Note. Results are on the response scale.
