# Supplementary material for: Active Gaze Guidance and Pupil Dilation Effects Through Subject Engagement in Ophthalmic Imaging
Source: J Eye Mov Res. 2025 Sep 19;18(5):45. doi: 10.3390/jemr18050045 (PMC12452769; doi:10.3390/jemr18050045)
Supplement: Supplementary file 1 [file jemr-18-00045-s001.zip › Pupil_Dilation.html]

JASP 


# Results

## Linear Mixed Models

| ANOVA Summary | | | | | | | |
| --- | --- | --- | --- | --- | --- | --- | --- |
| Effect | | df | | F | | p | |
| Condition |  | 1, 110.00 |  | 102.419 |  | < .001 |  |
| Pattern |  | 6, 110.00 |  | 6.310 |  | < .001 |  |
|  | | | | | | | |
|  |  |  |  |  |  |  |  |
| --- | --- | --- | --- | --- | --- | --- | --- |
| *Note.*  Model terms tested with Satterthwaite testMethod. | | | | | | | |
| *Note.*  The following variable is used as a random effects grouping factor: 'Subject'. | | | | | | | |
| *Note.*  Type III Sum of Squares | | | | | | | |

### Model summary

| Fit statistics | | | | | | | | | |
| --- | --- | --- | --- | --- | --- | --- | --- | --- | --- |
| Deviance (REML) | | log Lik. | | df | | AIC | | BIC | |
| 93.69 |  | -46.85 |  | 10 |  | 113.7 |  | 142.1 |  |
|  | | | | | | | | | |
|  |  |  |  |  |  |  |  |  |  |
| --- | --- | --- | --- | --- | --- | --- | --- | --- | --- |
| *Note.*  The model was fitted using restricted maximum likelihood. Please note that models with different fixed effects cannot be compared when REML is used. To use ML, switch 'Test method' to 'Likelihood ratio tests'. | | | | | | | | | |

| Sample sizes | | | |
| --- | --- | --- | --- |
|  | | Levels of RE grouping factors | |
| Observations | | Subject | |
| 126 |  | 9 |  |
|  | | | |

### Random Effect Estimates

| Subject: Random Effect Estimates | | | |
| --- | --- | --- | --- |
| Subject | | (Intercept) | |
| 1 |  | 2.970 |  |
| 2 |  | -0.746 |  |
| 3 |  | 0.727 |  |
| 4 |  | 1.528 |  |
| 5 |  | -0.374 |  |
| 6 |  | -0.671 |  |
| 7 |  | -0.423 |  |
| 8 |  | -1.090 |  |
| 9 |  | -1.921 |  |
|  | | | |

### Plot

| Estimated Marginal Means | | | | | | | | | |
| --- | --- | --- | --- | --- | --- | --- | --- | --- | --- |
|  | | | | | | 95% CI | | | |
| Condition | | Estimate | | SE | | Lower | | Upper | |
| Active |  | 5.898 |  | 0.500 |  | 4.918 |  | 6.878 |  |
| Passive |  | 5.432 |  | 0.500 |  | 4.452 |  | 6.412 |  |
|  | | | | | | | | | |
|  |  |  |  |  |  |  |  |  |  |
| --- | --- | --- | --- | --- | --- | --- | --- | --- | --- |
| *Note.*  Results are averaged over the levels of: Pattern. | | | | | | | | | |
