# Supplementary material for: Active Gaze Guidance and Pupil Dilation Effects Through Subject Engagement in Ophthalmic Imaging
Source: J Eye Mov Res. 2025 Sep 19;18(5):45. doi: 10.3390/jemr18050045 (PMC12452769; doi:10.3390/jemr18050045)
Supplement: Supplementary file 1 [file jemr-18-00045-s001.zip › Pupil_Dilation.pdf]

Results

Linear Mixed Models

ANOVA Summary

Effect	df	F	p
Condition	1, 110.00	102.419	< .001
Pattern	6, 110.00	6.310	< .001

Note. Model terms tested with Satterthwaite testMethod.

Note. The following variable is used as a random effects grouping factor: 'Subject'.

Note. Type III Sum of Squares

Model summary

Fit statistics

Deviance (REML)	log Lik.	df	AIC	BIC
93.69	−46.85	10	113.7	142.1

Note. The model was fitted using restricted maximum likelihood. Please note that models with different fixed effects cannot be compared when REML is used. To use ML, switch 'Test method' to 'Likelihood ratio tests'.

Sample sizes

	Levels of RE grouping factors
Observations	Subject
126	9

Random Effect Estimates

Subject: Random Effect Estimates

Subject	(Intercept)
1	2.970
2	−0.746
3	0.727
4	1.528
5	−0.374
6	−0.671
7	−0.423
8	−1.090
9	−1.921

Plot

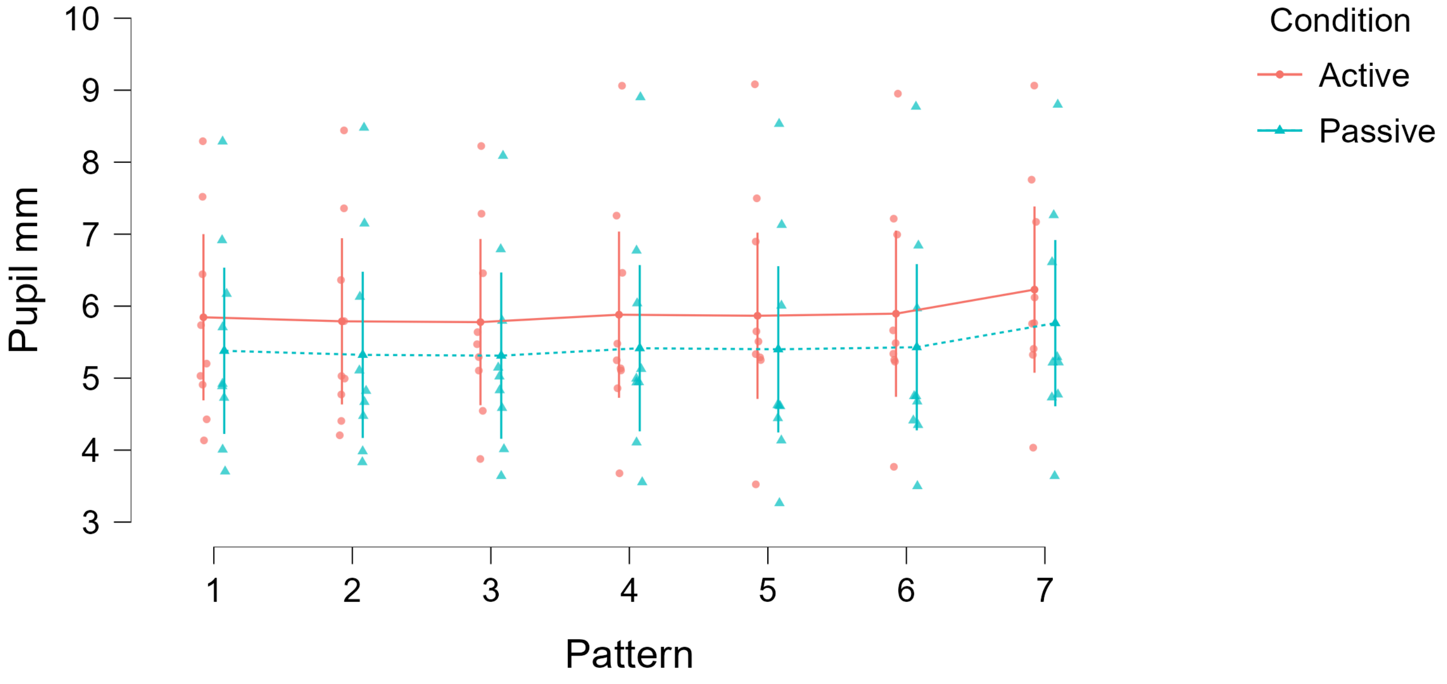

Estimated Marginal Means

			95% CI	
Condition	Estimate	SE	Lower	Upper
Active	5.898	0.500	4.918	6.878
Passive	5.432	0.500	4.452	6.412

Note. Results are averaged over the levels of: Pattern.
